# Comprehensive Comparative Molecular Characterization of Young and Old Lung Cancer Patients

**DOI:** 10.3389/fonc.2021.806845

**Published:** 2022-01-12

**Authors:** Mingming Hu, Jinjing Tan, Zhentian Liu, Lifeng Li, Hongmei Zhang, Dan Zhao, Baolan Li, Xuan Gao, Nanying Che, Tongmei Zhang

**Affiliations:** ^1^ Beijing Chest Hospital, Capital Medical University & Beijing Tuberculosis and Tumor Research Institute, Beijing, China; ^2^ Department of Tranlational Medicine, Geneplus-Beijing, Beijing, China; ^3^ State Key Laboratory of Microbial Resources, Institute of Microbiology, Chinese Academy of Sciences, Beijing, China; ^4^ Department of Tranlational Medicine, Geneplus-Shenzhen Clinical Laboratory, Shenzhen, China

**Keywords:** young lung cancer, NSCLC, prognosis, EGFR, molecular characteristics

## Abstract

**Background:**

Young lung cancer as a small subgroup of lung cancer has not been fully studied. Most of the previous studies focused on the clinicopathological features, but studies of molecular characteristics are still few and limited. Here, we explore the characteristics of prognosis and variation in young lung cancer patients with NSCLC.

**Methods:**

A total of 5639 young lung cancer samples (NSCLC, age ≤40) were screened from the SEER and the same number of the old (NSCLC, age ≥60) were screened by propensity score matching to evaluate the prognosis of two groups. 165 treatment-naïve patients diagnosed with NSCLC were enrolled to explore the molecular feature difference between two age-varying groups. CCLE cell line expression data was used to verify the finding from the cohort of 165 patients.

**Results:**

The overall survival of the young lung cancer group was significantly better than the old. Germline analysis showed a trend that the young group contained a higher incidence of germline alterations. The TMB of the young group was lower. Meanwhile, the heterogeneity and evolutionary degrees of the young lung cancer group were also lower than the old. The mutation spectrums of two groups exhibited variance with *LRP1B, SMARCA4, STK11, FAT2, RBM10, FANCM* mutations, EGFR L858R more recurrent in the old group and EML4-ALK fusions, BCL2L11 deletion polymorphism, EGFR 19DEL, 20IN more recurrent in the young group. For the base substitution, the young showed a lower fraction of transversion. Further, we performed a pathway analysis and found the EGFR tyrosine kinase inhibitor resistance pathway enriched in the young lung cancer group, which was validated in gene expression data later.

**Conclusions:**

There were significantly different molecular features of the young lung cancer group. The young lung cancer group had a more simple alteration structure. Alteration spectrums and base substitution types varied between two groups, implying the different pathogenesis. The young lung cancer group had more potential treatment choices. Although young lung patients had better outcomes, there were still adverse factors of them, suggesting that the young group still needs more caution for treatment choice and monitoring after the treatment to further improve the prognosis.

## Introduction

Lung cancer is the leading cause of cancer-related death in China and worldwide. According to previous reports, approximately 85% of patients are diagnosed with non-small cell lung cancer (NSCLC) (1). Though most NSCLC patients were with an average age of 70 when diagnosed, there were still 1% - 10% of patients younger than 40 years (2). The clinical characteristics showed that the young lung cancer group tended to be female, non-smoking, and with lung adenoma carcinomas (2, 3). And the young lung cancer group was often in the advanced stage when diagnosed (4). The prognosis varied between the young lung cancer group and the old lung cancer group after receiving the same treatment. Despite the relatively advanced stage of the young lung cancer group, previous studies showed a generally better prognosis of the young lung cancer group compared with the old lung cancer group (4–7).

Although there are many comparative studies on lung cancer between young and old people, most of them are from the clinicopathological perspective. Despite some reports finding the related features of young cancer patients like EGFR mutation, ROS1, and ALK rearrangement (6, 7), studies that examined molecular alterations characteristics of the young population were still scarce and limited to the somatic level. To reveal the molecular landscape of the young lung cancer group, a comprehensive comparative analysis of the young lung cancer group and the old lung cancer group are still necessary to be carried out. According to the description of old age from WHO (The World Health Organisation) and other studies of young lung cancer, and to aim at the effect of age, we defined lung cancer patients aged ≥60 as the old lung cancer group and lung cancer patients aged ≤ 40 as the young lung cancer group (2–7).

In our study, we performed a survival analysis with data from SEER to explore the prognosis difference between the two groups. Then molecular characteristics of two groups were analyzed at the germline level first. A following somatic-level analysis was performed, exploring the difference of SNVs, CNVs, base substitutions, and pathways.

## Materials and Methods

### Clinical Cohort

In this retrospective cohort study, 165 patients diagnosed with NSCLC at Beijing Chest Hospital were enrolled. The clinical characteristics of all patients were summarized in **Table 1**. This study was approved by Beijing Chest Hospital affiliated to Capital Medical University Ethics Committee. All participants provided informed written consent before undergoing any study-related procedures. This study was performed in accordance with the Declaration of Helsinki.

**Table 1:** Clinical information of the cohort with 165 lung cancer patients.

	Age<=40 yr, N (%)	Age>=60 yr, N (%)	P
Gender			<0.001
Male	24 (32.9%)	66 (71.7%)	
Female	49 (67.1%)	26 (28.3%)	
Histology			<0.001
Adenocarcinoma	42 (57.5%)	71 (77.2%)	
Squamous	4 (5.5%)	18 (19.6%)	
Unknown	27 (37%)	3 (3.3%)	
Staging			<0.001
I	22 (30.1%)	19 (20.7%)	
II	7 (9.6%)	17 (18.5%)	
III	13 (17.8%)	55 (59.8%)	
IV	9 (12.3%)	0 (0%)	
Unknown	22 (30.1%)	1 (1.1%)	

### Sample Collection and DNA Extraction

Tumor tissue was sampled *via* surgery. All patients were treatment-naïve when sampling. Formalin fixation and paraffin embedding were then performed, followed by histologic section preparation. Genomic DNA was isolated from FFPE tumor samples using the QIAamp DNA FFPE Tissue Kit (Qiagen GmbH, Hilden, Germany), according to the manufacturer’s protocol. The DNA concentration was measured using the Qubit dsDNA HS (High Sensitivity) assay kit in the Qubit fluorometer (Invitrogen; Thermo Fisher Scientific, Inc., Waltham, MA, USA). To test the DNA integrity, 200 ng extracted DNA was loaded onto the 1% agarose gel with λ -Hind III digest DNA marker (Takara Biotechnology Co., Ltd., Dalian, China). The DNA samples that were longer than the second largest bonds (9,416 bp) of λ -Hind III digest DNA marker were considered as integrated samples and used for subsequent analysis.

### Library Preparation

Tumor DNA was sheared into 200-250-bp fragments using a Covaris S2 instrument (Woburn, MA, USA), and indexed NGS libraries were prepared using the DNA Library Preparation Kit for MGISeq-2000 (BGI, Shenzhen, China). Additional detailed information regarding library preparation was described by Lv et al. (8).

### Target Region Capture and Next-Generation Sequencing

All libraries were hybridized to custom-designed biotinylated oligonucleotide probes (IDT, Coralville, IA, USA) covering 1021 genes (exonic coverage of 0.96 Mb). All included genes are shown in **Table S1**. DNA sequencing was performed using the MGISeq-2000 Sequencing System (BGI, Shenzhen, China) per the manufacturer’s guideline, which generated 3 Gb of data from tumor DNA. Additional detailed information regarding target region capture and NGS was described by Lv et al (8).

### Raw Data Processing

After removing raw reads containing adaptor sequences, those with more than 50% low-quality base reads, or those with more than 50% N bases reads were mapped to the reference human genome (GRCh37) using the Burrows-Wheel Aligner (http://bio-bwa.sourceforge.net/) with default parameters. Duplicate reads were identified and marked with Picard’s Mark Duplicates tool (https://software.broadinstitute.org/gatk/documentation/tooldocs/4.0.3.0/picard_sam_markduplicates_MarkDuplicates.php) for tumor DNA data. Errors introduced by PCR or sequencing were corrected according to clustered reads. Local realignment and base quality recalibration were performed using The Gene Analysis Toolkit (https://www.broadinstitute.org/gatk/).

### Somatic Mutation Calling of Tumor DNA

Somatic single-nucleotide variations (SNVs) were called using the MuTect2 algorithm (https://software.broadinstitute.org/gatk/documentation/tooldocs/3.8-0/org_broadinstitute_gatk_tools_walkers_cancer_m2_MuTect2.php). Candidate mutations were filtered if (1) The allele frequency was less than 1%; (2)Variants were filtered as cross-contamination if present in >0.1% samples in single nucleotide polymorphism (SNP) databases (dbsnp, https://www.ncbi.nlm.nih.gov/projects/SNP/; 1000G, https://www.internationalgenome.org/; ESP6500, https://evs.gs.washington.edu/; ExAC, http://exac.broadinstitute.org/). (3) The SIFT score >0.05 or PolyPhen2 score <0.85 but keep harmful mutations that can cause disease. The final candidate variants were all manually verified in the Integrative Genomics Viewer (IGV) and the remaining mutations were considered validated somatic variants (9).

### Pathway Enrichment Analysis

The online database metascape (http://metascape.org) was used to conduct pathway and process enrichment analysis. In our study, the Gene Ontology (GO) terms for biological process, Kyoto Encyclopedia of Genes and Genomes (KEGG) pathways, Reactome Gene Sets, Canonical Pathways, and PANTHER Pathway were enriched based on the Metascape online tool. Only terms with the P-value < 0.01, minimum count of 3, and enrichment factor of >1.5 were considered as significant.

### SEER Database

In this study, To compare the difference in prognosis between youth and elderly, we analyzed the SEER (The Surveillance, Epidemiology, and End Results, http://seer.cancer.gov) database NSCLC cases from 1975 to 2018. SEER is representative of the US population, with patient-level data abstracted from 18 geographically diverse populations that represent rural, urban, and regional populations. A total of 363,342 samples and matched clinical information were included in the analysis, of which 5639 samples age <=40 samples and 357,703 samples age >=60 samples. To minimize the effects of potential confounders in the analysis, the 1:1 nearest neighbor propensity score matching (PSM) method was implemented using Stage, Sex, Race, and Grade as confounding variables by MatchIt R program packages.

### CCLE Database

We download 32 CCLE (Cancer Cell Line Encyclopedia) NSCLC samples (treatment naïve) from the CCLE database (https://portals.broadinstitute.org/) (22460905), of which 7 cell lines age <=40 and 15 cell lines age >=60. Differential expression analysis was performed with clusterProfiler package (10) and KEGG pathway enrichment analysis was performed with Metascape online tool.

### PyClone Analysis

PyClone was used to analyze the clonal population structure of tumor samples from each patient (11). PyClone infers the clonal composition of a tumor by grouping single nucleotide variation (SNV) with similar cell frequencies together. Variants located in the cluster with the greatest mean cancer cell fraction (CCF) were defined as clonal and the rest were subclonal.

### MATH Determination

The MATH value of each allele was calculated from the median absolute deviation (MAD) and the median of its mutant AFs: MATH = 148.26 × MAD/median. The key purpose of the MATH value is to reflect the fluctuation range of AFs in the same sample and can be used as a measure of genomic heterogeneity (12).

### Statistical Analysis

Fisher’s exact test was used to compare categorical variables. The Kaplan-Meier method with the log-rank test was used to calculate the probability of OS. The effect of risk factors on OS was evaluated by the Cox proportional hazards regression model. All statistical analyses and presentations were performed using R v4.0. Statistical significance was set at p < 0.05.

## Results

### Clinical Characteristics of Patients

The basic clinical information was exhibited in **Table 1**. The fraction of female patients in the young lung cancer group was significantly higher than the old (Fisher exact test, p<0.0001). There was a trend that the young lung cancer group contained more adenocarcinoma compared with the old lung cancer group (Fisher exact test, p=0.067). The staging structure was also different between the two groups.

### Prognosis Difference between the Young Lung Cancer Group and the Old Lung Cancer Group

**Figure 1:**
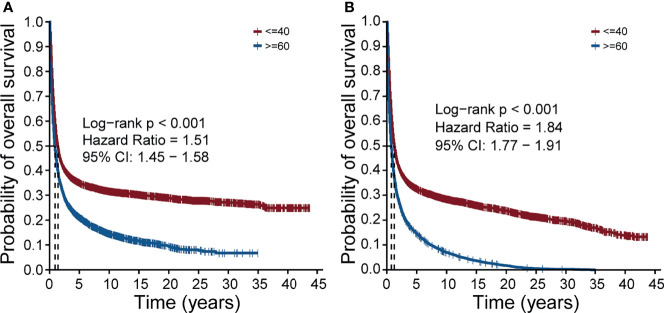
Overall survival of the young lung cancer group and the old lung cancer group in the SEER cohort. **(A)** The overall survival of patients with cancer-specific death. **(B)** The overall survival of patients with non-accidental death.

To confirm whether there was a prognosis difference between two groups separated by age, a total of 5639 young lung cancer samples (age ≤40) were screened from the SEER and the same number of old lung cancer samples (age ≥60) were screened with PSM. Then we performed survival analysis and the result showed that regardless of cancer-specific death (**Figure 1A**) or non-accidental death (**Figure 1B**), the prognosis of the young lung cancer group was significantly better than that of the old (both p values < 0.001, Log-rank test; hazard ratios = 1.51, 1.84, respectively). This trend remained when evaluating the prognosis in the cohort without the PSM (**Figures S1A, B**).

### Comparison of Germline Genome Characteristics between the Young Lung Cancer Group and the Old Lung Cancer Group

**Figure 2:**
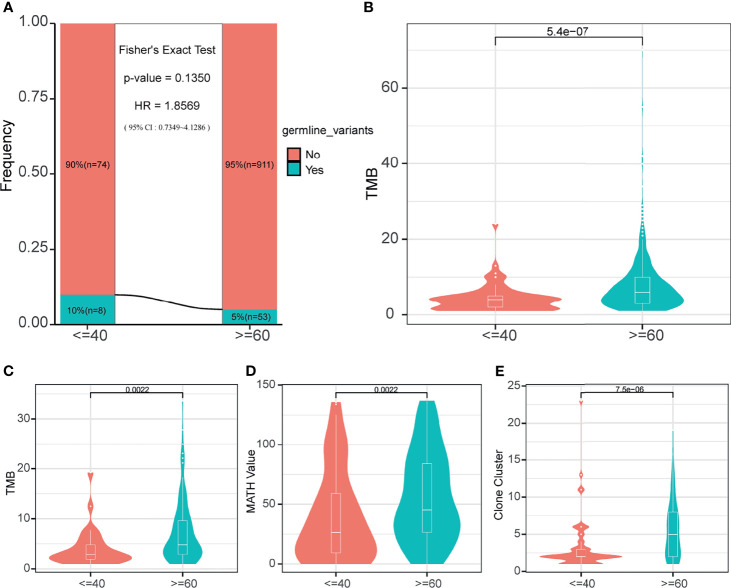
Germline mutation status and somatic mutation characteristics. **(A)** Germline mutation frequency of two age-varying groups in the cohort with germline mutation information. **(B)** The TMB of patients in the cohort with germline mutation information. **(C)** The somatic TMB of patients in the 165-sample cohort. **(D)** MATH values of patients in the 165-sample cohort. **(E)** The number of clone clusters of patients in the 165-sample cohort.

Since the prognosis difference had been confirmed, we tried to find the difference in the molecular level between the two groups, which may affect the prognosis. Because of the low diagnostic age of the young lung cancer group, normally we consider them as the susceptible population. Therefore the germline genome features are necessary to be explored between the two groups. Firstly, it is needed to make clear whether the existence of germline mutations was related to young lung cancer. We explored in a cohort of 1046 lung cancer patients with germline mutation data. The results showed there was the trend that the fraction of patients with the germline mutation was higher in the young lung cancer group (n = 8,10%) than in the old lung cancer group (n=53, 5%) (**Figure 2A**, p = 0.135, HR =1.8569). Then the TMB of patients was compared between two groups, and it was found that the TMB of this cohort in the young lung cancer group was significantly lower than the old lung cancer group (**Figure 2B**, p < 0.0001).

### Comparison of Somatic Genome Characteristics between the Young Lung Cancer Group and the Old Lung Cancer Group

For the somatic level, the following genome characteristics analysis was performed in our cohort. Comparing the TMB (**Figure 2C**), MATH value (**Figure 2D**), and Clone cluster number (**Figure 2E**) values between the young lung cancer group and the old lung cancer group, we found that the values in the young lung cancer group were all significantly lower.

**Figure 3:**
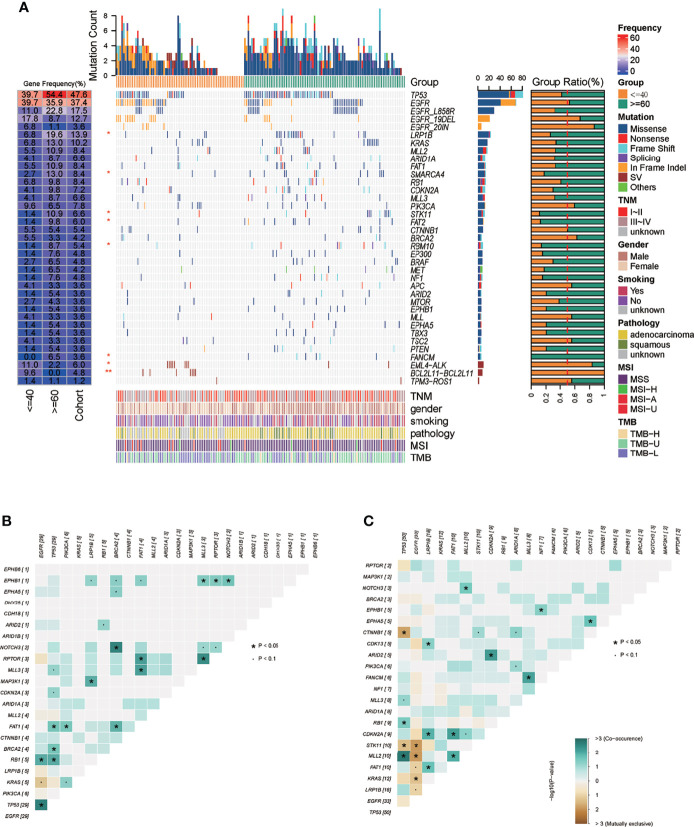
Mutation landscape of the 165-sample cohort. **(A)** Mutation spectrum of young and old lung cancer groups in the 165-sample cohort. **(B)** The mutually concurrent and exclusive genes in the young lung cancer group. **(C)** The mutually concurrent and exclusive genes in the old lung cancer group. "*" means the p value < = 0.05.

By comparative analysis of the genetic landscape, we found that the mutation frequency of *LRP1B, SMARCA4, STK11, FAT2, RBM10, FANCM* genes in the old lung cancer group was significantly higher than that in the youth group, while EML4-ALK fusion, BCL2L11 deletion polymorphism in the young group was higher than that in the old group (**Figure 3A**, Fisher’s Exact Test, p ≤ 0.05). Specific to EGFR, there was a trend that the incidence of EGFR 19DEL, 20 IN in the young group was higher than that in the old group, while the mutation frequency of the EGFR L858R mutation was opposite (**Figure 3A**). For the aspect of mutually concurrent and exclusive genes, in the young lung cancer group, there were genes significantly concurrent with actionable driver genes (EGFR, KRAS, ARID1A: TP53 and RB1 with EGFR; PIK3CA with KRAS and ARID1A (**Figure 3B**). In the old lung cancer group, there were genes significantly concurrent with actionable driver genes (CDKN2A, NF1): FAT1, LRP1B, ARID2 with CDKN2A; EPHB1 with NF1, and genes significantly exclusive with actionable driver genes (EGFR, KRAS): MLL2, STK11, KRAS with EGFR (**Figure 3C**). Notably, there were three significantly concurrent pairs in the young lung cancer group (EGFR with concurrent RB1 and TP53; KRAS with concurrent PIK3CA) showed mutually exclusive in the old lung cancer group (**Figures 3B, C**).

**Figure 4:**
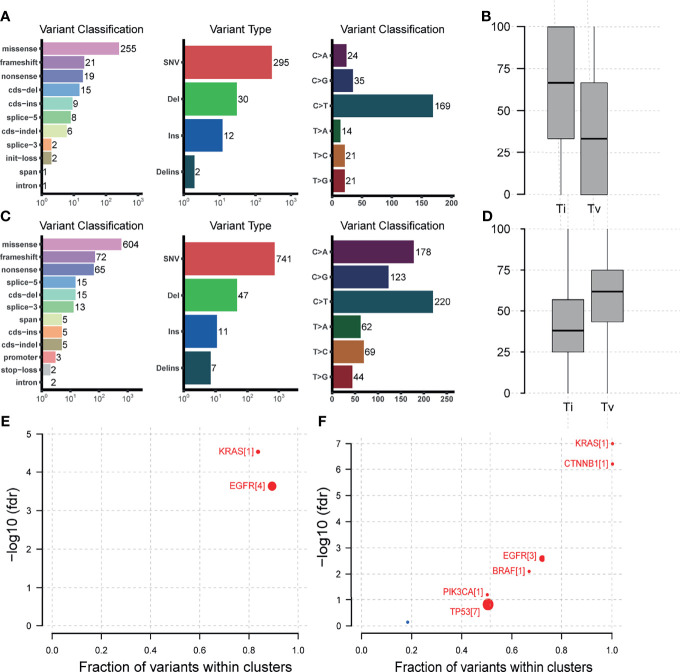
Mutation types, base substitution types, and driver gene status in the 165-sample cohort. **(A)** Mutation types, base substitution types of the young lung cancer group. **(B)** Transition and transversion fractions of the young lung cancer group. **(C)** Mutation types, base substitution types of the old lung cancer group. **(D)** Transition and transversion fractions of the old lung cancer group. **(E)** Driver gene status of the young lung cancer group. **(F)** Driver gene status of the old lung cancer group.

As to the detailed somatic mutation types, there was no obvious difference between the two groups, with missense dominating the variant classification and SNV dominating the variant type in both groups (**Figures 4A, C**). While the status of base substitution types was not the same. The most base substitution type in the young group was C>T transition (n=169, 59.5%) which was significantly higher than in the old (n = 220, 31.6%, p= 0.0019), with the fraction of other types nearly the same (**Figure 4A**). In the old group, the most base mutation type was still C>T transition, but with C>A transversion, C>G transversion also occupying most of the base mutations (**Figures 4A, C**, n=178, 25.6%, n=123, 17.7%). For base substitution types, it is noted that in the young group, transitions were much more than transversions (**Figure 4B**, p = 0. 00023), but in the old group, in contrast, transversions were much more than transitions (**Figure 4D**). Detailed variant type and substitution information was shown in **Figure S2**. Furthermore, we found that KRAS and EGFR were driver genes in the young group (**Figure 4E**). In the old group, except KRAS and EGFR, CTNNB1, BRAF, PIK3CA, TP53 were also the driver genes (**Figure 4F**).

**Figure 5:**
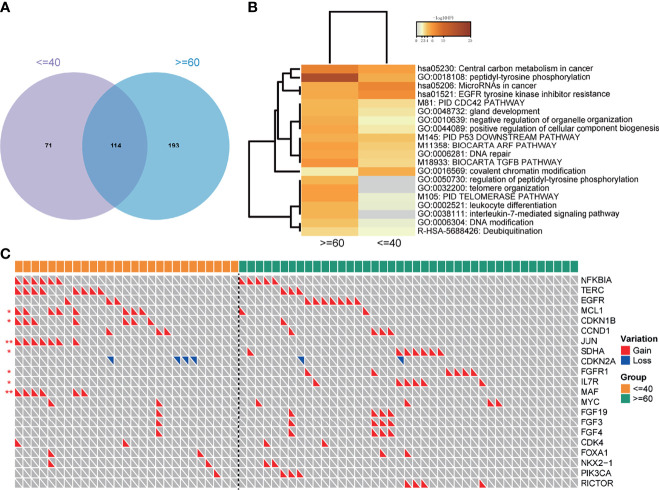
Pathway analysis and the distribution of CNVs in the 165-sample cohort. **(A)** The number of overlapped mutations in the cohort. **(B)** Pathways that significantly enriched in two groups. **(C)** CNVs landscape of two groups. "*" means the p value < = 0.05; "**" means the p value < = 0.01.

In general, the young group and the old group had 114 shared mutations (**Figure 5A**), with the unique mutations of 71 (38.4%) and 193 (62.9%), respectively, revealing the moderate difference of mutation types between the two groups.

To explore the genome characteristics of two groups deeply, a pathway analysis was performed. Notably, the EGFR tyrosine kinase inhibitor resistance pathway was significantly enriched in the youth group (**Figure 5B**), implying that the young lung cancer group was easier to get EGFR-TKI resistance. Besides, the Covalent chromatin modification was also enriched in the young lung cancer group. However, the peptidyl-tyrosine phosphorylation, DNA repair, DNA modification pathways were enriched in the old lung cancer group (**Figure 5B**).

The CNVs status between the two groups was also compared. The prevalences of gains of MCL1, CDKN1B, JUN, MAF were significantly higher in the young group than that in the old group. On the contrary, the prevalences of gains of SDHA, FGFR1, and IL7R were significantly lower in the young group. As to EGFR, the prevalence of CNVs in the young group was lower than that in the old group (11.1% vs 17.1%), but without statistical significance (**Figure 5C**).

### Clonality and Actionability of EGFR Mutations in the Young Group

**Figure 6:**
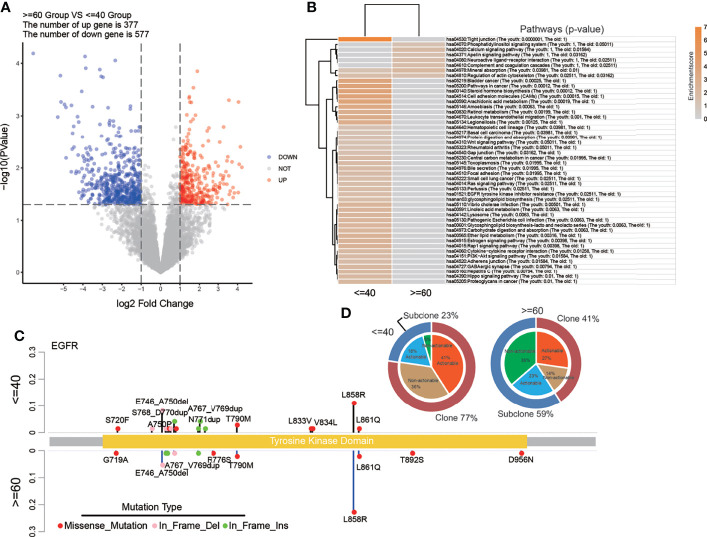
Pathway analysis with RNA data from CCLE and detailed EGFR mutation status of the 165-sample cohort. **(A)** The volcano plot of CCLE RNA data showing the differentially expressed genes. **(B)** Pathways that significantly enriched in two groups with CCLE RNA data. **(C)** The lollipop plot showed the distribution of EGFR mutation types. **(D)** The clonality and actionability of EGFR alterations in the 165-sample cohort.

Further, we applied a pathway analysis with the KEGG gene set using the CCLE cell line DNA expression data. The number of up genes was 377 and the number of the down gene was 577 (**Figure 6A**). And it was notable that the EGFR tyrosine kinase inhibitor resistance pathway was significantly enriched in the young group, but not in the old group, which was consistent with the above pathway analysis with genome data (**Figure 6B**). Then the distribution of EGFR alterations was counted in a lollipop plot (**Figure 6C**), which illustrated the phenomenon that the number of EGFR mutation types in the young group was larger than that in the old group, but the prevalence of EGFR L858R was lower. The proportion of clonal EGFR mutations were higher in the young group than in the old group (77% *vs* 41%), and meanwhile, the proportion of clonal actionable mutations was also higher (41% *vs* 27%, **Figure 6D**).

## Discussion

In our study, we started with the survival analysis of two age-varying groups. Then to investigate the molecular features behind this, we compared the genome characteristics of two groups in germline level and the following somatic level including the SNVs, CNVs, TMB, heterogeneity, base substitution, and evolutionary path. Given the EGFR-TKI resistance pathway enriched in the young group, we validated the result in a gene expression level with CCLE cell line data and compared the EGFR variance between the two groups in detail.

Due to the lack of prognosis information in our cohort, we performed the survival analysis with the matched data from the SEER database by the PSM method. As expected, the results showed significant variance that the young cancer group had better outcomes compared with the old lung cancer group, which was consistent with previous studies (4–7). Even though the young lung cancer group showed adverse clinical features such as advanced tumor stages, they still had advantages on the prognosis. One reason may be that the prognosis of patients was influenced by the treatment therapy to a great extent. The young lung cancer patients tended to receive a more radical therapy, which may cause a better prognosis for them. However, treatment information was not supplied by the SEER. This problem might be solved after we get the prognosis results from the patients of our cohort in the future.

As cancer is a disease that needs to accumulate enough genomic alterations and because of the relatively early age of the young lung cancer group, we speculated that the young lung cancer group may contain germline advantages of oncogenesis. Therefore in the subsequent molecular analysis, firstly we evaluated the germline alteration incident of two groups. The result exhibited the trend that the young lung cancer group had a higher germline alterations incidence compared with the old lung cancer group, indicating the hereditary susceptibility of the young lung cancer patients.

The subsequent somatic analysis also exhibited a lower TMB level in the young lung cancer group. Oncogene-addicted NSCLCs usually exhibit a lower TMB (13–15). And young lung cancer patients harboring more oncogene driver mutations, which may explain this phenomenon that the TMB level in the young lung cancer group was lower. Moreover, the relatively lower TMB suggested that the young lung cancer group may obtain fewer benefits from immunotherapy (16, 17).

And the MATH value reflecting the heterogeneity of tumors in young cancer patients was also lower. Besides, the clone cluster number of the young lung cancer group was larger than the old, which suggested the shorter evolutionary pathway. Following the pattern of development of tumors, the somatic alteration characteristics showed above were all consistent with the lower age of the young lung cancer group and demonstrated the relatively simple alteration structure in the young lung cancer group (18).

As for the genetic landscape in detail, several incidences of mutated genes that were adverse to prognosis were observed to be lower in the young lung cancer group (*LRP1B, SMARCA4, STK11, FAT2, RBM10, FANCM)*, which may explain the better prognosis of the young lung cancer group to some extent. But it is more notable that the EML4-ALK fusion in the young group was higher than that in the old group. The EML4-ALK gene fusion was detected in only 4–8% of lung cancers mainly in light smokers or nonsmokers (19), and was the known driver factor of lung cancer (20). While in the young lung cancer group, the incidence of EML4-ALK was 11.0%, which was much higher. That may be the reflection of the oncogenesis feature of the young lung cancer group. The BCL2L11 deletion polymorphism in the young group was also higher than that in the old group. The previous study has reported that patients with BCL2L11 deletion polymorphism got a relatively poor efficacy from the osimertinib, which has the directive significance of treatment selection to the young lung cancer group (21). For co-occurring genes and exclusive genes, three gene pairs showed opposite status between two groups. In the following study, together with the prognosis information of our cohort, we will explore how this molecular pattern difference affected the prognosis of the two groups.

The base substitution pattern of mutations reflects the biological background of the mutation genesis. In the young group, transitions were much more than transversions, but in the old group, transversions were more than transitions. The phenomenon of transversion-high is strongly associated with smoking (22). The C>T transition was the critical characteristic of mutation signature 6 which is associated with defective DNA mismatch repair. And this type of substitution was significantly more in the young lung cancer group. Those results implied the different pathogenesis of lung cancer between the two groups.

When taking the driver gene into account, the results of MAFtools showed a more complex constitution of driver genes in the old group with KRAS, EGFR, CTNNB1, BRAF, PIK3CA, and TP53 compared to the young group with KRAS and EGFR only, which was in accordance with the relatively simple tumor genetic evolutionary pattern of the young lung cancer group.

We have mentioned the low heterogeneity of the young lung cancer group. Nevertheless, when considering the EGFR alone, we found that the EGFR alterations in the young group were more diverse than that in the old group. Both the overall clonal EGFR mutation fraction and the actionable EGFR mutation fraction of the young group were higher than those of the old group. These features implied potentially more choices of EGFR-TKIs in young lung cancer treatment. While in the pathway analysis, the EGFR tyrosine kinase inhibitor resistance pathway was identified in the young group. Then we validated this pathway by cell line gene expression data from the CCLE and the same enrichment result was observed, suggesting the more probable formation of drug resistance of the young lung cancer group to EGFR-TKIs. And a previous study demonstrated that younger age was associated with lower EGFR-TKIs efficacy (23), which was consistent with the enrichment of the EGFR tyrosine kinase inhibitor resistance pathway. EGFR 20IN alterations in the young lung cancer group were much enriched. Besides, there were two types of drug: amivantamab(mOS = 22.8 mth) and mobocertinib (mOS = 24 mth), receiving Accelerated Approval from the FDA for the treatment of advanced-stage non-small-cell lung cancer patients with EGFR exon 20 INs in 2021, which brought new choice for young lung cancer patients. And more drugs and combination approaches for patients with EGFR exon 20 INs are under investigation (24). However, a recent study reported that the EGFR exon 20 mutation was heterogeneous in its response to TKIs, some of which were pan-sensitive to EGFR TKIs, while EGFR 20IN-L was only sensitive to second-generation TKI (25), which suggested that the young lung cancer group still needed more caution when treatment selection and when monitoring after receiving EGFR-TKIs treatment.

For other pathways that were variant between two groups, it was notable that covalent chromatin modification was significantly enriched in the young lung cancer group. It mainly refers to histone modifications, including acetylation, methylation, phosphorylation, adenylation, ubiquitination, ADP ribosylation, etc. At present, deacetylase inhibitors and demethylase inhibitors have been applied in clinical practice and drug development (26). A previous study has reported that targeting EHMT2 can reverse EGFR-TKI resistance in NSCLC at the epigenetic level (27).

A previous study showed that the overall survival of young lung cancer patients was better than the old. However, this advantage tended to be marginal for advanced young lung cancer patients with stages III and IV (28). Because of the limited number of patients, we did not analyze the molecular characteristics according to the different stages of patients. In the following study, we plan to enlarge the cohort and study the molecular characteristics and mechanisms in detailed aspects. Our study contained limitations of the lack of prognosis and treatment information. Moreover, most of the results were generated from genomic data. In the future, prospective studies of a larger cohort with comprehensive clinical information are still needed to make clear the clinicopathology and multi-omics features of young lung cancer patients and to explore the potential correlation between those features and patients’ prognosis. Thus treatment strategies more specific to the young lung cancer patients can be developed to get better clinical outcomes.

## Conclusions

In our study, it was shown that the prognosis of the young lung cancer group was significantly better than that of the old lung cancer group. There was a trend that the young lung cancer group kept a higher occurrence rate of germline mutation. The young lung cancer group had a more simple alteration structure with lower heterogeneity and a shorter evolution path. Small variations, base substitution types, and CNVs varied between two age-varying groups, revealing the difference pathogenesis between them. The fact that both clonality and actionability of EGFR in the young lung cancer group were higher than those in the old lung cancer group, and the covalent chromatin modification pathway enriched in the young lung cancer group implied the multiple choices of young lung cancer treatment. Although young lung patients had better outcomes and many molecular features of them predicted a good prognosis, however, there were still the EGFR-TKI resistance pathway and BCL2L11 deletion polymorphism, as well as diverse EGFR 20 insertions, which may have an adverse influence on those patients, suggesting that the young group still needs more caution for treatment choice and monitoring after the treatment, and this may further improve the prognosis of young lung cancer patients.

## Data Availability Statement

The datasets presented in this study can be found in online repositories. The names of the repository/repositories and accession number(s) can be found below: [GSA (Genome Sequence Archive): HRA001658]. Link: https://ngdc.cncb.ac.cn/gsa-human/browse/HRA001658.

## Ethics Statement

The studies involving human participants were reviewed and approved by the Beijing Chest Hospital affiliated to Capital Medical University Ethics Committee. The patients/participants provided their written informed consent to participate in this study.

## Author Contributions

NC and TZ conceptually designed the work and supervised the whole study. HZ, BL, and DZ collected the patient’s samples together with matched clinical information and performed the pathological tests. XG and LL carried out the NGS sequencing and realize data visualization. MH, JT, and ZL performed the data collection, data analysis and drafted the manuscript. All authors contributed to the article and approved the submitted version.

## Funding

This work was supported by Beijing Municipal Science and Technology Commission Z171100001017038, Tongzhou Lianggao Talents Project YH201920, Tongzhou District Science and Technology Committee Project KJ2020CX010 to TZ.

## Conflict of Interest

ZL, LL, and XG are employees of Beijing-Geneplus Technology Limited.

The remaining authors declare that the research was conducted in the absence of any commercial or financial relationships that could be construed as a potential conflict of interest.

## Publisher’s Note

All claims expressed in this article are solely those of the authors and do not necessarily represent those of their affiliated organizations, or those of the publisher, the editors and the reviewers. Any product that may be evaluated in this article, or claim that may be made by its manufacturer, is not guaranteed or endorsed by the publisher.
