# Supplementary figures and images for: Comprehensive Comparative Molecular Characterization of Young and Old Lung Cancer Patients
Source: Front Oncol. 2022 Jan 12;11:806845. doi: 10.3389/fonc.2021.806845 (PMC8789686; doi:10.3389/fonc.2021.806845)

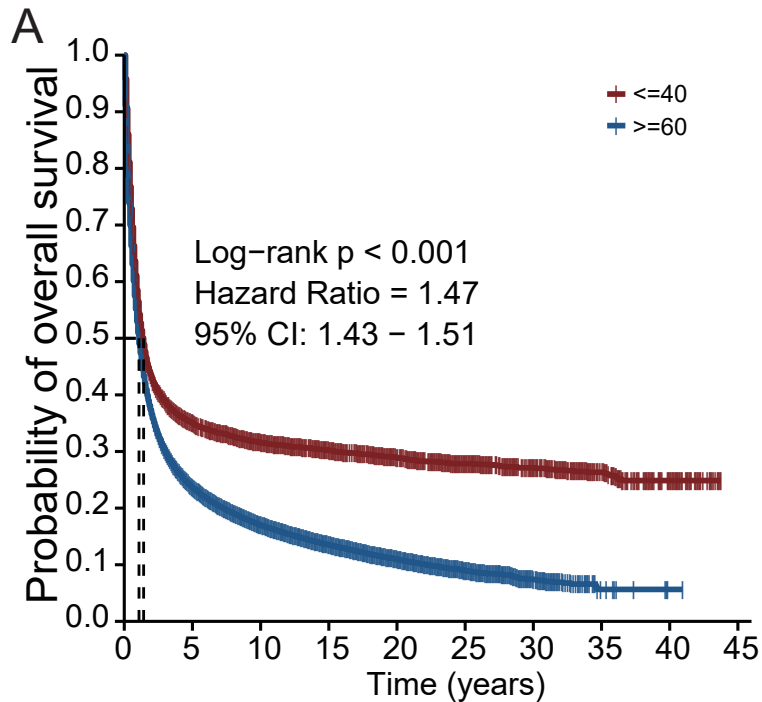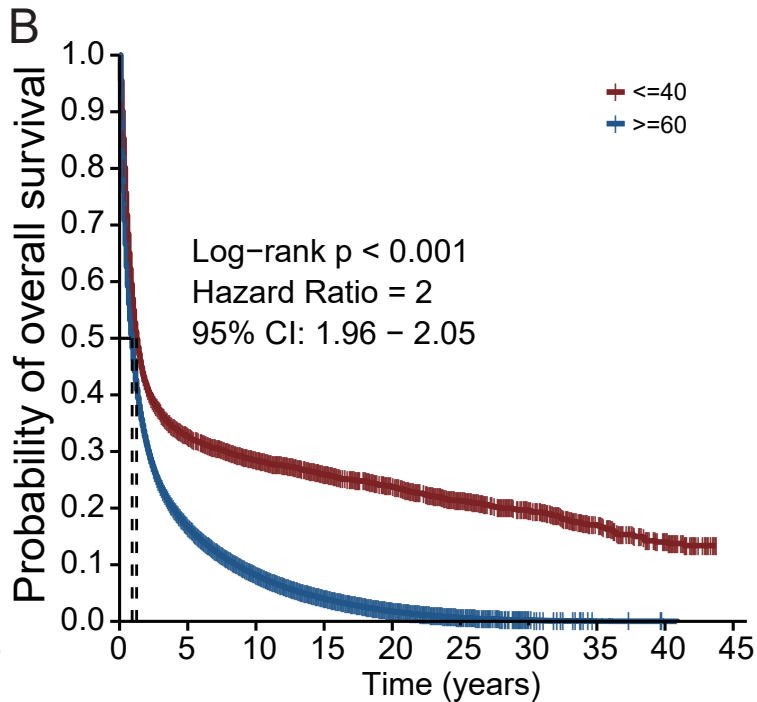

Supplement: Supplementary file 1 [file DataSheet_1.pdf]

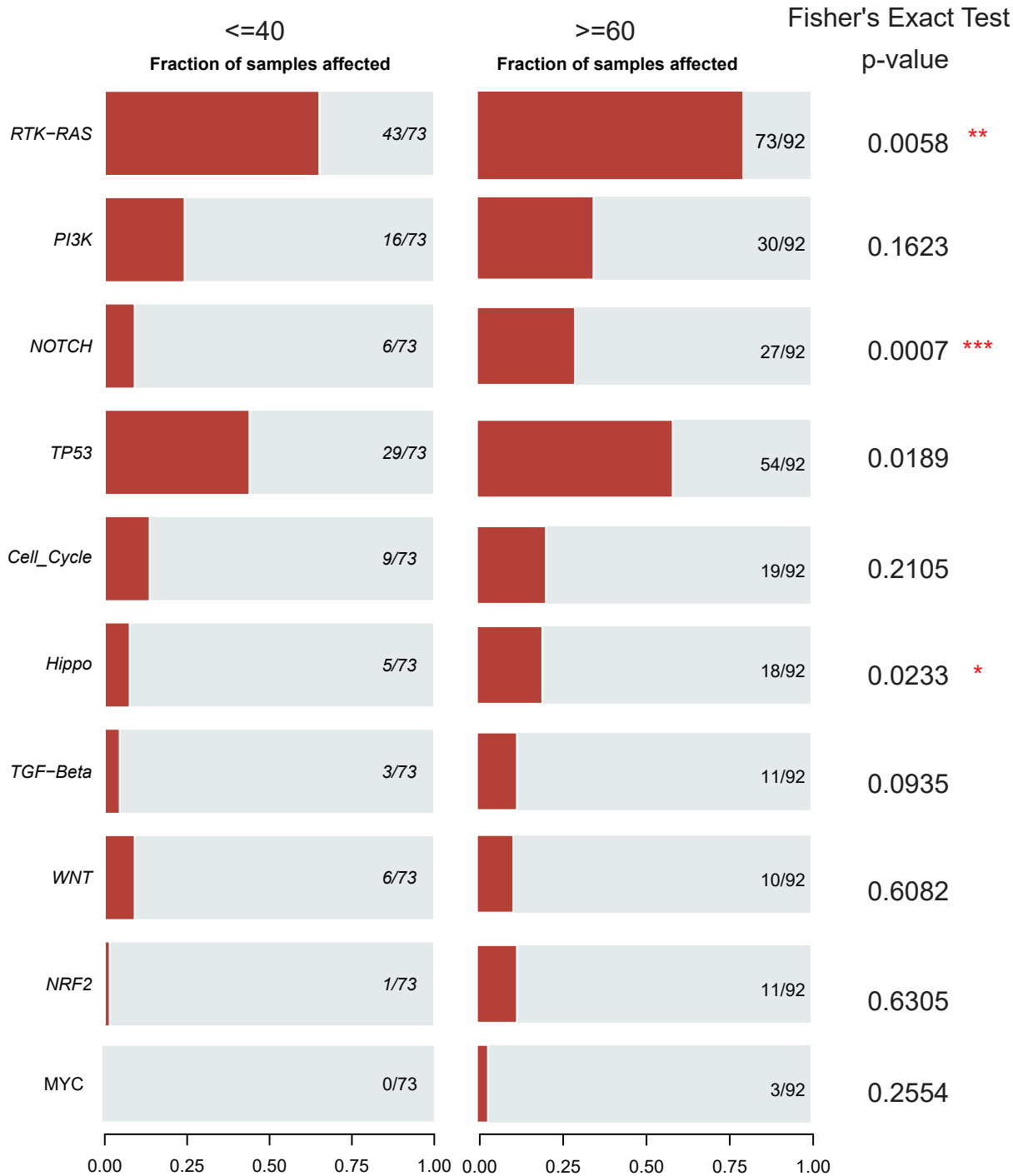

Supplement: Supplementary file 2 [file DataSheet_2.pdf]

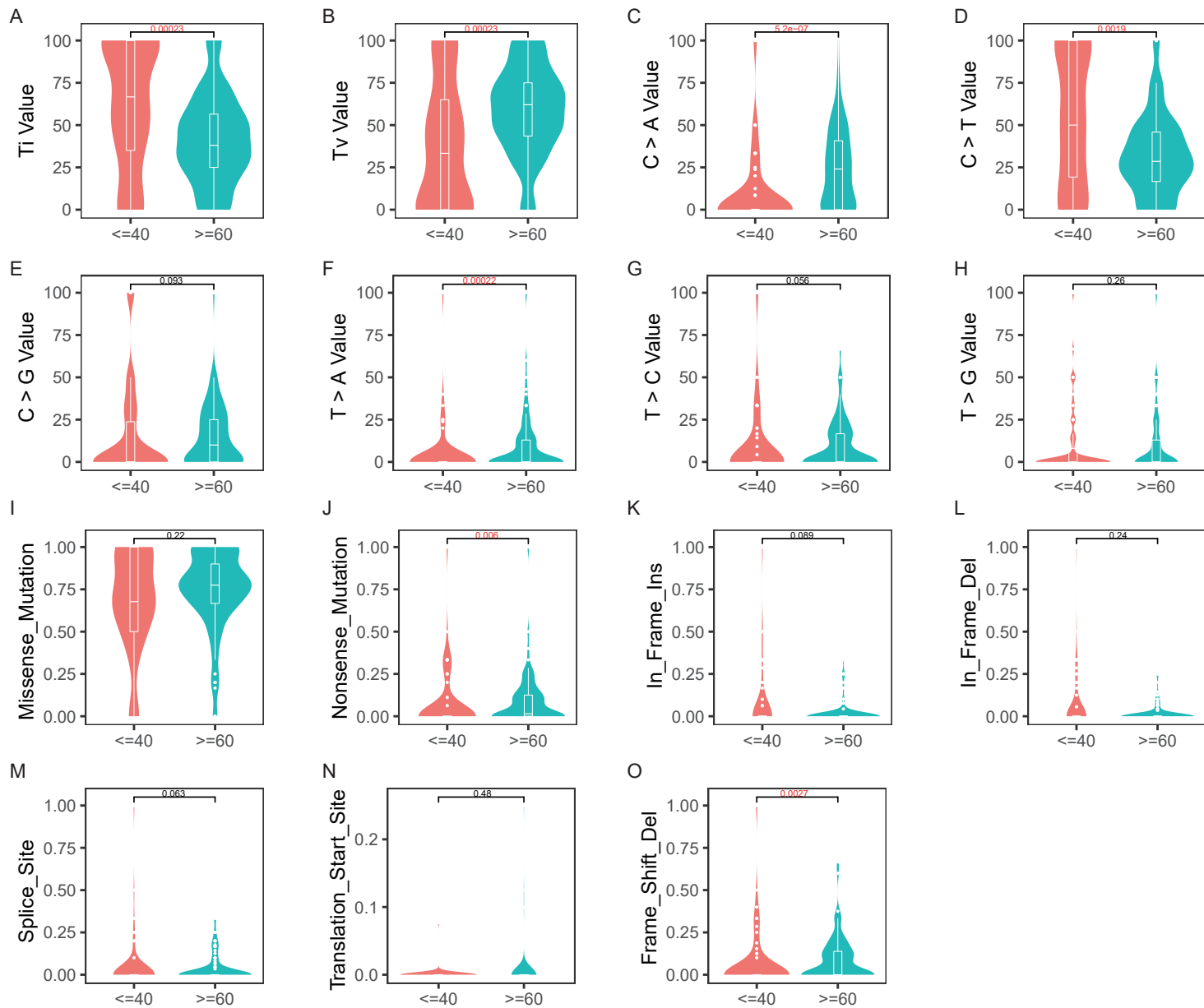

Supplement: Supplementary file 3 [file DataSheet_3.pdf]
